# Shenling Baizhu Decoction (SLBZD) may play a synergistic role of tirelizumab in the treatment of colorectal cancer by influencing the imbalance of colon flora and Tumor microenvironment

**DOI:** 10.7150/jca.88854

**Published:** 2024-01-01

**Authors:** Xue Deng, Chunxia Zhang, Yang Yang, Jun Wang, Xiaorui Ye, Jiabo Gu, Xinyi Zhang, Heiying Jin

**Affiliations:** Department of Colorectal Surgery, The Second Affiliated Hospital of Nanjing University of Chinese Medicine,23Nanhu Road, Nanjing 210017, China.

**Keywords:** colorectal cancer, tirelizumab, Shenling Baizhu Decoction (SLBZD), colon flora, tumor microenvironment (TME)

## Abstract

**Objective:** To explore whether SLBZD can play a synergistic role in promoting the efficacy of PD-1 inhibitors in the treatment of colorectal cancer by influencing the intestinal microenvironment and Tumor microenvironment.

**Method:** Shenling Baizhu Decoction (SLBZD) and tirelizumab (TLzmab) treated the colorectal mouse model. The tumor growth rate, tumor weight, and tumor growth inhibition rate were evaluated. Fecal microbiota was detected by 16S rDNA sequencing and immune cell was detected by the flow cytometry analysis.

**Result:** Compared to tumor weight, there exist significant differences between each group among the three groups. Compared to tumor volume, there was no statistically significant difference in tumor size between the control group and the TLzmab group at 7 days. However, there was a statistical difference in tumor size among the three groups at 18 days. By analyzing the diversity of the Gut microbiota, the diversity decreased after TLzmab treatment with a statistically significant difference. Compared with the control group, the diversity of the TLzmab+SLBZD group increased. The proportion of lymphocytes in the blood was analyzed by flow cytometry. Compared with the control group, Myeloid-derived suppressor cells (MDSCs) decreased and T regulatory cells (Treg) increased significantly in the TLzmab group. Compared with the control group and TLzmab group, the TLzmab+SLBZD group showed a significant increase in M1 type macrophages, while the M2 type macrophages, MDSCs, and Treg showed a significant decrease.

**Conclusion:** An imbalance of Gut microbiota and imbalance of tumor immune microenvironment will occur during TLzmab treatment, which will lead to poor therapeutic effect of TLzmab or drug resistance. SLBZD will increase the abundance of Gut microbiota, which will lead to the increase of M1 macrophages in the tumor immune microenvironment and the decrease of M2 macrophages and Treg cells, thus exerting the synergistic effect of TLzmab. This study provides a new way to explore the improvement of ICIs through traditional Chinese medicine.

## Introduction

The morbidity and mortality rates of colorectal cancer rank third and second respectively in malignant tumors [1.2]. Recurrence and metastasis are still important factors leading to the death of colorectal cancer patients. Immune checkpoint inhibitors (ICIs) have unique efficacy for advanced CRC 3. Cercek *et al.* treated 12 locally advanced rectal cancer patients with MSI-H with a PD-1 inhibitor (dostarlimab) every three weeks for a total of 6 months and found that all patients had complete clinical remission without further surgery or chemotherapy. No tumor recurrence was found during the follow-up period of 6 to 25 months 4. Hu *et al.* applied PD-1 inhibitors (toripalimab) for preoperative neoadjuvant treatment of locally advanced or stage Ⅲ colorectal cancer with MSI-H and found that 88% of patients had complete remission postoperatively 5.

However, in colorectal cancer, only 15-20% of microsatellite highly unstable/mismatch repair defects (MSI-H/dMMR) patients are sensitive to ICIs, while 80-85% of colorectal cancer patients do not respond to colorectal cancer patients. Even colorectal cancer that is sensitive to ICIs treatment may develop resistance after a period of treatment 6. Therefore, addressing the issues of drug resistance and low sensitivity in ICIs treatment is a key issue in improving the treatment of colorectal cancer with ICIs.

The efficacy of ICIs is closely related to Tumor microenvironment (TME) and gut microbe. TME is composed of tumor cells, immune cells, fibroblasts, Myeloid-derived suppressor cells (MDSCs), and various Signaling molecule, which has an important impact on the development of tumors [7.8]. Among them, regulatory T cells (Tregs), Tumor-associated macrophages (TAMs), and MDSCs can mediate immunosuppression in a variety of ways, leading to drug resistance in ICIs 9. The gut microbe can influence the immune response, thereby directly affecting the efficacy of immunotherapy 10.

Studies have found that the use of antibiotics can cause intestinal flora dysbiosis, significantly reducing the effect of PD-1 antibody 11. The change in intestinal flora homeostasis is an important factor affecting the TME. Studies showed that fecal bacteria transplantation (FMT) can improve the tumor immune microenvironment by improving the intestinal m flora, and play a synergistic role in ICIs. Routy *et al.* 12 found that microbiota dysbiosis could promote tumor cell immune escape, leading to immunotherapy resistance. Baruch *et al.* 13 found that 10 malignant Melanoma patients with PD-1/PD-L1 immunotherapy resistance received fecal microbiome transplantation (FMT), one of whom was completely relieved, and two of whom were partially relieved. The three effective patients had immune cell infiltration in the intestinal Lamina propria and TME. By regulating the Gut microbiota, the efficacy of ICIs can be restored or promoted. Davar *et al.* 14 reported that 15 Melanoma patients with PD-1/PD-L1 immunotherapy resistance received FMT, and 5 patients benefited clinically and induced rapid and lasting microbiota reconstruction. However, the use of FMT is not very convenient, and the clinical efficacy will also decrease as time goes on. It is of important clinical significance to find drugs that can improve the Gut microbiota to play a synergistic effect on ICIs.

Shenling Baizhu Decoction (SLBZD) is derived from the "Taiping Huimin He Ji Ju Fang", a classic prescription for “strengthening the spleen and promoting dampness” dating back 1000 years from the Song Dynasty. Its main function is often applied to inflammatory bowel disease, colorectal tumors and irritable bowel syndrome. In our previous study, it was found that Shenling Baizhu Decoction (SLBZD) can inhibit the recurrence of colorectal polyps after polypectomy, and can improve the structure of intestinal Gut microbiota. After SLBZD administration, the B:F value of Gut microbiota increased from 0.196 to 0.275, and the detection of bacteria species indicated that rumen bacteria, Dorea, Clostridium R and other bacteria groups increased significantly compared with those before administration, suggesting that the SLBZD can improve the structure of Gut microbiota, thus playing a role in preventing colorectal polyps 15. Pharmacological studies have found that SLBZD contains compounds such as Quercetin, kaempferol, stigmasterol, etc., which can play an anti-tumor role through anti-inflammatory, immune regulation, repair of intestinal mucosal damage, regulation of intestinal microbiota structure *et al.* 16-17. Therefore, this study aims to explore whether SLBZD can play a role in promoting the efficacy of PD-1 inhibitors in the treatment of colorectal cancer by influencing the intestinal microenvironment and Tumor microenvironment. The study was proved by the ethics committee of The Second Affiliated Hospital of Nanjing University of Chinese Medicine (2022SEZ-041).

## Methods

### 1. Preparation of the experimental drug

The SLBZD is composed of 10 traditional Chinese medicinal herbs: 20g of Ginseng, 20g of White Atractylodes, 20g of Poria, 20g of Licorice, 10g of Coix Seed, 9g of Amomum, 15g of Hyacinth Bean, 20g of Chinese Yam, 10g of Balloon Flower Root, and 10g of Lotus Seed. All herbs were purchased from the Traditional Chinese Medicine Dispensary of the Second Hospital of Jiangsu Province (Nanjing, China), with a total of 15 batches weighing 2310g.

Firstly, the Chinese medicinal herbs were soaked in 75% ethanol solution at 8 times their volume overnight. Then, the filtrate was collected after vacuum drying for 1.5 hours. Subsequently, the herbs were processed with 75% ethanol solution at 6 times their volume for 1 hour, and the filtrate was collected. After vacuum concentration for 4 hours, the extract was obtained, collected, and dried at 60°C, resulting in a dry extract powder.

The dosage of the experimental drug was determined based on clinical medication dosage and adjusted according to the body surface area rate of mice relative to humans, with a final dosage of 0.5g/kg/d.

To improve the solubility of the dry extract powder in the solvent, 0.5% sodium carboxymethyl cellulose (CMC-Na) was used. The dry extract powder was accurately weighed and then suspended in 0.5% CMC-Na, resulting in a final concentration of 0.5g/mL for the traditional Chinese medicinal drug.

Tisleizumab (TLzmab): Batch Number S20190045, from Bioprofile China Ltd.

### 2. Establishment of Subcutaneous Tumor Model of Colorectal Cancer in Mice according to literature reports 18

In this study, a total of 24 male BALB/c-Hpd1 mice with a weight of 16±2g and SPF grade, aged 4 weeks, were purchased from Nanjing Laboratory Animal Center (animal certificate number: NO.11400700154060, experimental animal production license number: SCXK (Su) 2011-0003)). After one week of acclimation under specific pathogen-free conditions, HCT116 cells were cultured routinely at 5% CO2 and 37°C. After transfection and selection, 1×10^6^ cells (0.2ml) were taken and subcutaneously inoculated in the right neck skin of each mouse after disinfection with 0.5% iodine. The mice were then fed regularly for approximately 3 weeks.

On days 7, 14, and 18 after the inoculation, the tumor images were observed and captured under a live fluorescence imaging system. The long and short diameters of the tumors were measured, and the tumor volume (V) was calculated as V = (tumor long diameter × tumor short diameter²) / 2. When the tumor formation rate of the mice reached 100% and the subcutaneous tumor volume was ≥1cm³, the mice were randomly divided into three groups:

Control group(G1): On days 1 and 7, mice were intraperitoneally injected with 4ml of Normal Saline; from days 1 to 7, mice were administered 2ml of Normal Saline by gavage.

PD-1 monoclonal antibody group (G2): On days 1 and 7, mice were intraperitoneally injected with 200μg of TLzmab (50μg/ml); from days 1 to 7, mice were administered 2ml of physiological saline by gavage.

SLBZD combined with PD-1 monoclonal antibody group(G3): On days 1 and 7, mice were intraperitoneally injected with 200μg of TLzmab (50μg/ml); from days 1 to 7, mice were administered 1g/day (0.5g/ml) of SLBZD by intragastric administration, and observation continued until the 18th day after drug administration.

The tumor long and short diameters of all mice were measured daily using a caliper. Tumor volume was estimated using the formula: length × width² × 0.5.

The tumor growth inhibition rate (TGI, %) was calculated as [(average tumor volume of the control group - average tumor volume of the treated group) / average tumor volume of the control group] × 100%.

The tumor weight inhibition rate (TWI, %) was calculated as [(average tumor weight of the control group - average tumor weight of the treated group) / average tumor weight of the control group] × 100%.

### 3. Analysis of Mouse Fecal Microbiota

On the 18th day after the start of the treatment, fresh fecal samples from mice were collected and immediately transferred to a -80°C freezer for storage. The samples were then transported to Dongwo Rui Biotechnology, and dry ice was used during transportation to maintain their integrity. The Gut microbiota was analyzed using 16S rDNA sequencing. Genomic DNA was extracted from the samples using the CTAB method. Specific primers with barcodes (515F-806R targeting the V4 region of the 16S rRNA gene) were used for PCR amplification. The PCR products with a size range of 400-450bp, as determined by gel electrophoresis, were mixed and subjected to library construction using the Illumina TruSeq DNA PCR-Free Library Preparation Kit. After quantification and library quality control with Qubit, qualified libraries were sequenced using the NovaSeq 6000 platform to generate raw data. After data processing, including assembly and filtering, valid data were obtained.

The valid data were then used for operational taxonomic units (OTUs) clustering and taxonomic classification analysis. Based on the OTUs clustering results, the representative sequences of each OTU were taxonomically annotated to obtain corresponding species information and their abundance distribution. QIIME 2 platform was used for further analysis, including abundance calculation of OTUs, alpha diversity, and beta diversity calculations. The results were visualized through PCoA (Principal Coordinates Analysis) plots and sample clustering trees.

LEfSe statistical analysis method was employed to assess the differential abundance of species and community results between different groups of samples.

### 4. Flow Cytometry

On the 18th day after the start of the treatment, mice were euthanized, and peripheral blood was collected from the orbital venous plexus using EDTA-Li microcapillary tubes for minimal anticoagulation. The blood was stained with KIRAVIA Blue 520™ anti-mouse F4/80 Antibody (Cat # 123161, BioLegend, USA), APC/Fire™ 750 anti-mouse/human CD11b Antibody (Cat # 101261, BioLegend, USA), APC anti-mouse CD86 Antibody (Cat # 105011, BioLegend, USA), PE anti-mouse CD206 (MMR) Antibody (Cat # 141705, BioLegend, USA), PE anti-mouse CD25 Antibody (Cat # 113703, BioLegend, USA), Alexa Fluor® 647 anti-mouse FOXP3 Antibody (Cat # 126407, BioLegend, USA), FITC anti-mouse CD4 Antibody (Cat # 100405, BioLegend, USA) and PE/Cyanine7 anti-mouse Ly-6G/Ly-6C (Gr-1) Antibody (Cat # 108415, BioLegend, USA) for flow cytometry analysis. The flow cytometry analysis was performed on a Fortessa flow cytometer (BD Biosciences, San Jose, CA). Data analysis was conducted using FlowJo version 10 software (Tree Star Inc., Ashland, OR). The antibodies of immune cells for flow cytometer detection are listed in Table 1.

### 5. Data Analysis

Statistical analysis was carried out using SPSS version 26.0 (SPSS, Chicago, IL, USA). Quantitative data were expressed as mean ± standard deviation. Independent sample t-test was used for statistical analysis between two groups, and one-way ANOVA was used for statistical analysis among multiple groups. A significance level of p<0.05 was considered statistically significant.

## Results

### 1. SLBZD significantly improves the efficacy of TLzmab treatment of colorectal cancer

**Figure 1:**
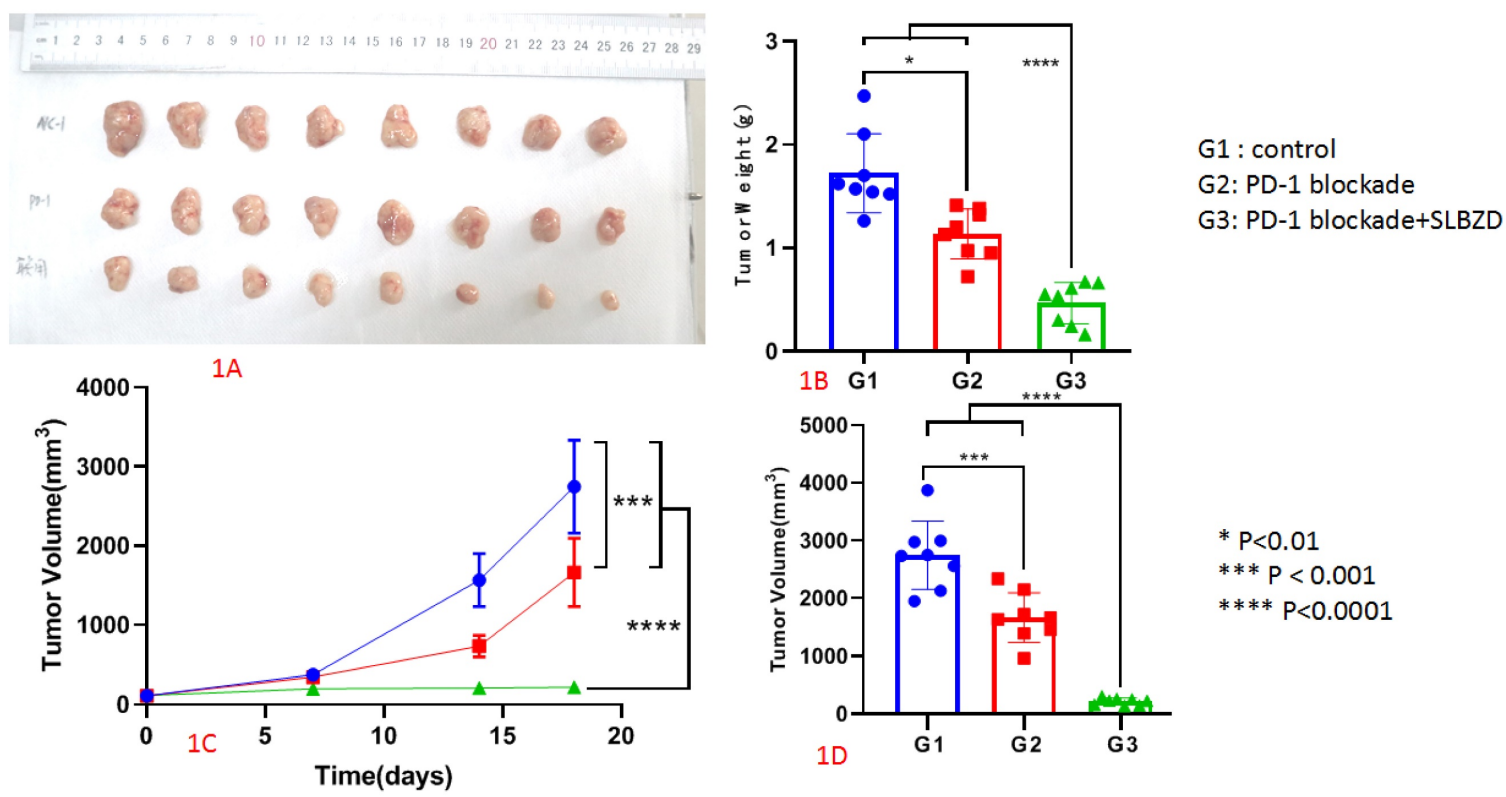
The growth of tumor after treatment byPD-1 blockade and SLBZD (1A: Tumor in 3 groups;1B Tumor weight in 3 groups;1C: tumor growth curve in 3 groups;1D: Tumor volume in 3 groups).

In this study, we observed the therapeutic effects using a xenograft mouse model. The tumor weigh was 1.72 ± 0.38g in the control group (G1), 1.14 ± 0.24g in the TLzmab group (G2), and 0.47 ± 0.20g in the SLBZD+TLzmab group (G3) respectively.One Way ANOVA was performed on the tumor weight and multiple comparisons were performed to compare the two groups. The control group vs. TLzmab group (p<0.01), control group vs. SLBZD+TLzmab group (P<0.001), and TLzmab group vs. SLBZD+TLzmab group (P<0.001) showed significant differences (Fig. 1A, 1B).

The tumor volume was recorded in Table 1. One-way ANOVA was performed on the tumor volume of mice at 0, 7, 14, and 18 days, and multiple comparisons were performed between the groups. There was no significant difference in tumor size between the groups at 0 days (p=0.68), and there was no statistically significant difference in tumor size between the control group and the TLzmab group at 7 days (p=0.345). However, there was a statistically significant difference between SLBZD+TLzmab and the control group (p<0.0001) and SLBZD group (p<0.001). There was a statistical difference in tumor size among the three groups at 18 days (P<0.0001), while there was a significant difference in SLBZD+TLzmab group and TLzmab group (p<0.001) (Fig. 1C, 1D). The tumor volume in 3 groups on day 0, day 7, day 14, and day 18 were listed in Table 2.

**Figure 2:**
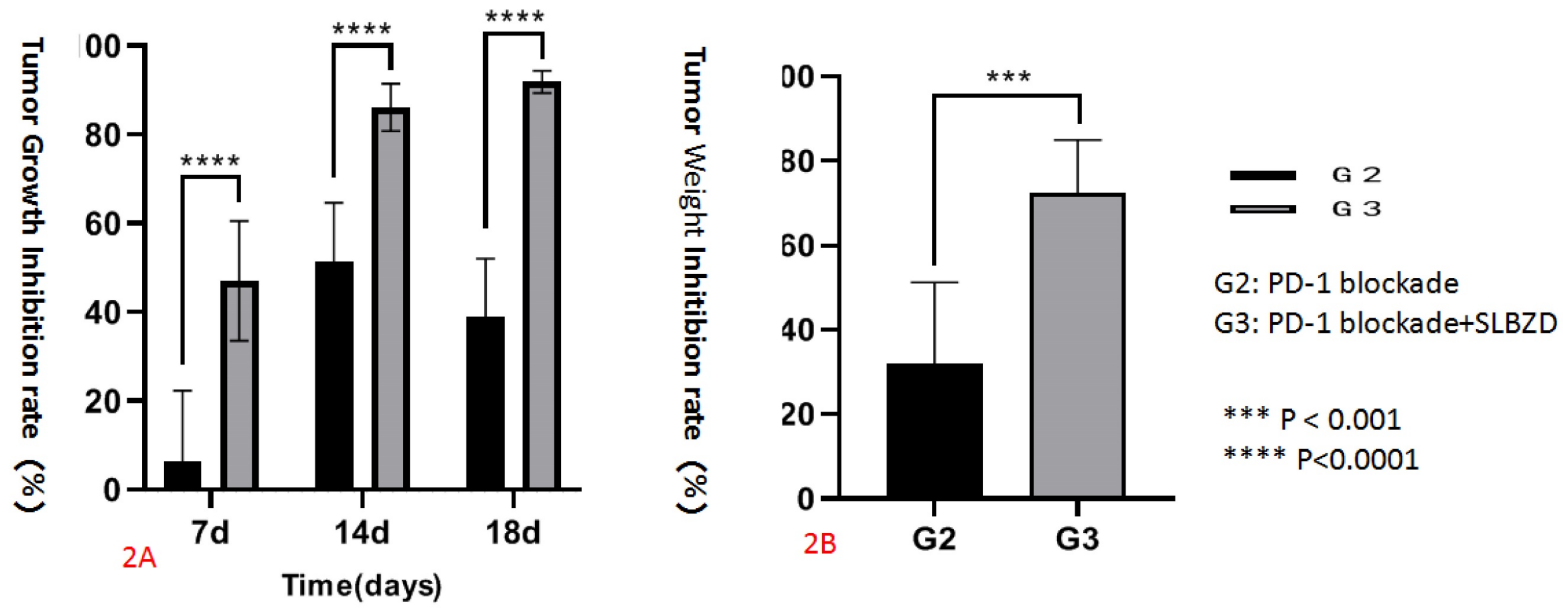
The tumor inhibition rate in G2 and G3 (2A: Tumor Growth Inhibition rate; 2B Tumor Weight Inhibition rate).

The tumor growth volume inhibition rates (TGI) on the 7th, 14th, and 18th days were 7.68%, 53.16%, 39.36% (TLzmab group), 47.28%, 86.80%, and 92.09% (SLBZD+TLzmab group), respectively, indicating that SLBZD can achieve more ideal and lasting therapeutic effects in PD-1 monoclonal antibody immunotherapy (P<0.001); The tumor weight inhibition rates (%) at 18 days were 34.11% in the TLzmab group and 73.00% in the SLBZD+TLzmab group, respectively (p<0.001). (Figure 2).

### 2. Regulation of SLBZD on Gut microbiota in mice

**Figure 3:**
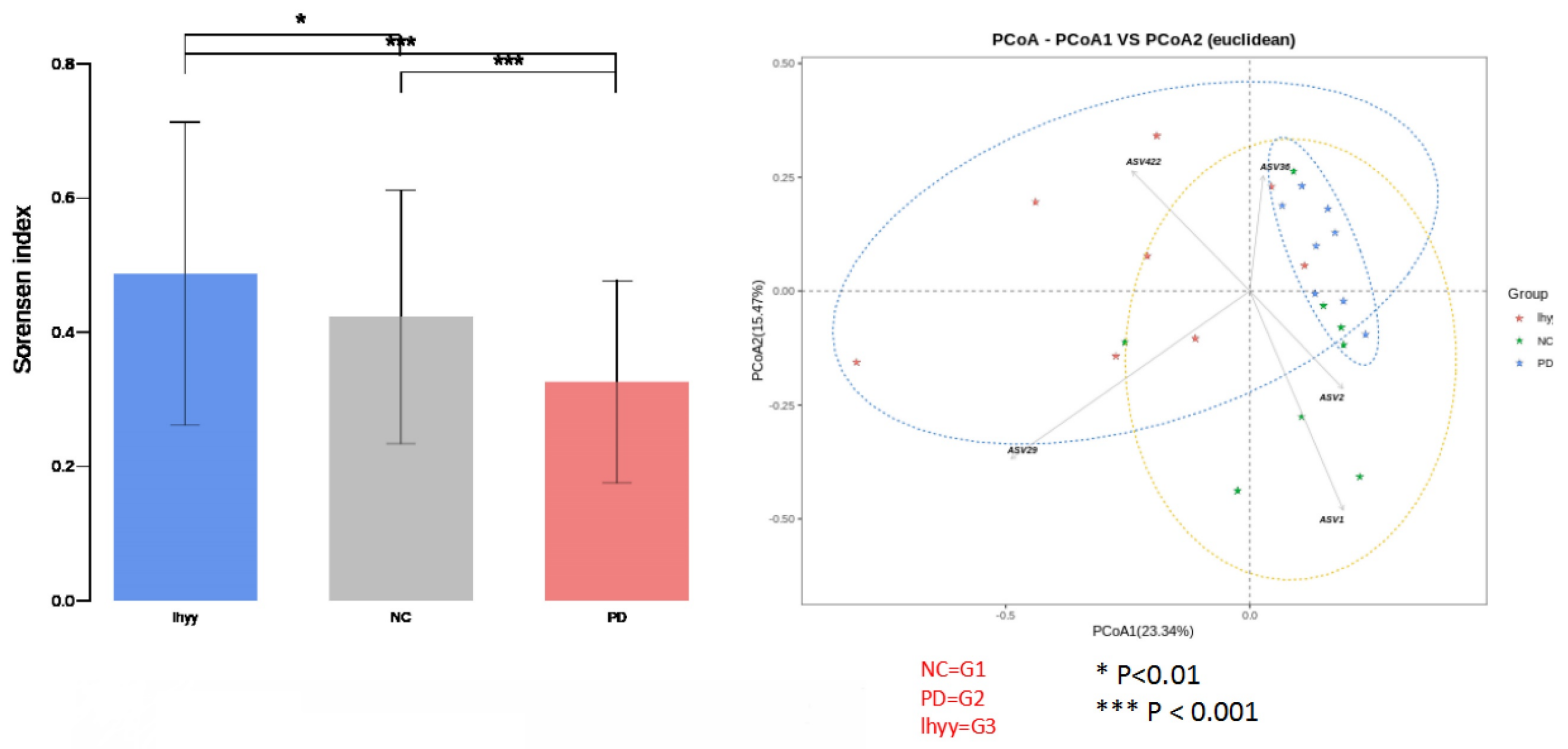
The beta abundance and PCA analysis in 3 groups.

By analyzing the diversity of the Gut microbiota of the three groups of mice, it was found that compared with the control group, the beta diversity decreased after TLzmab treatment with a statistically significant difference (p<0.001). Compared with the control group, the beta diversity of the TLzmab+SLBZD group increased, with a statistically significant difference (P<0.01). Compared with the TLzmab group, the beta diversity of the TLzmab+SLBZD group increased more significantly, with a significant difference (p<0.001), suggesting that TLzmab can reduce the diversity of Gut microbiota after treatment and increase the diversity of Gut microbiota after using the SLBZD. Through the PcoA diagram, we found that the flora community of the three groups of samples was significantly separated, and the composition of the Gut microbiota community of the SLBZD combined group mice was significantly different from the other two groups, indicating that SLBZD had a regulatory effect on the structure of the Gut microbiota of mice (Figure 3).

**Figure 4:**
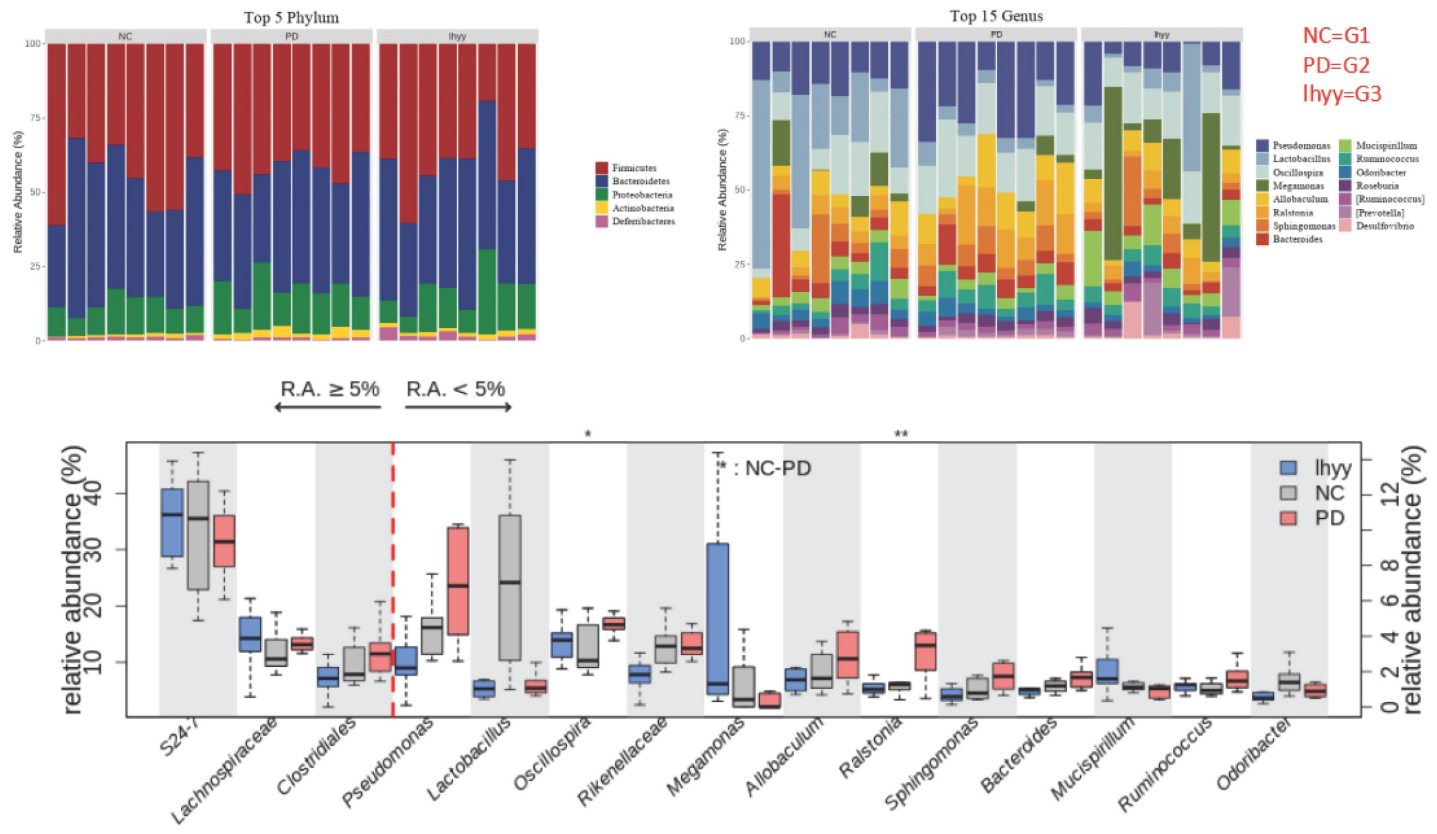
The abundance of the gut microbe in 3 groups.

At the phylum level, the first five categories of the three groups are Bacillota, Bacteroidota, Pseudomonadota, Actinomycetota, and Deferribacteraceae. At the genus level, 24_ 7 (Muribagulaceae), Spirillum, Megamonas, Spirillum, Pseudomonas, Clostridium, Sphingomonas, and Bacteroides have significant changes in relative abundance (Figure 4).

**Figure 5:**
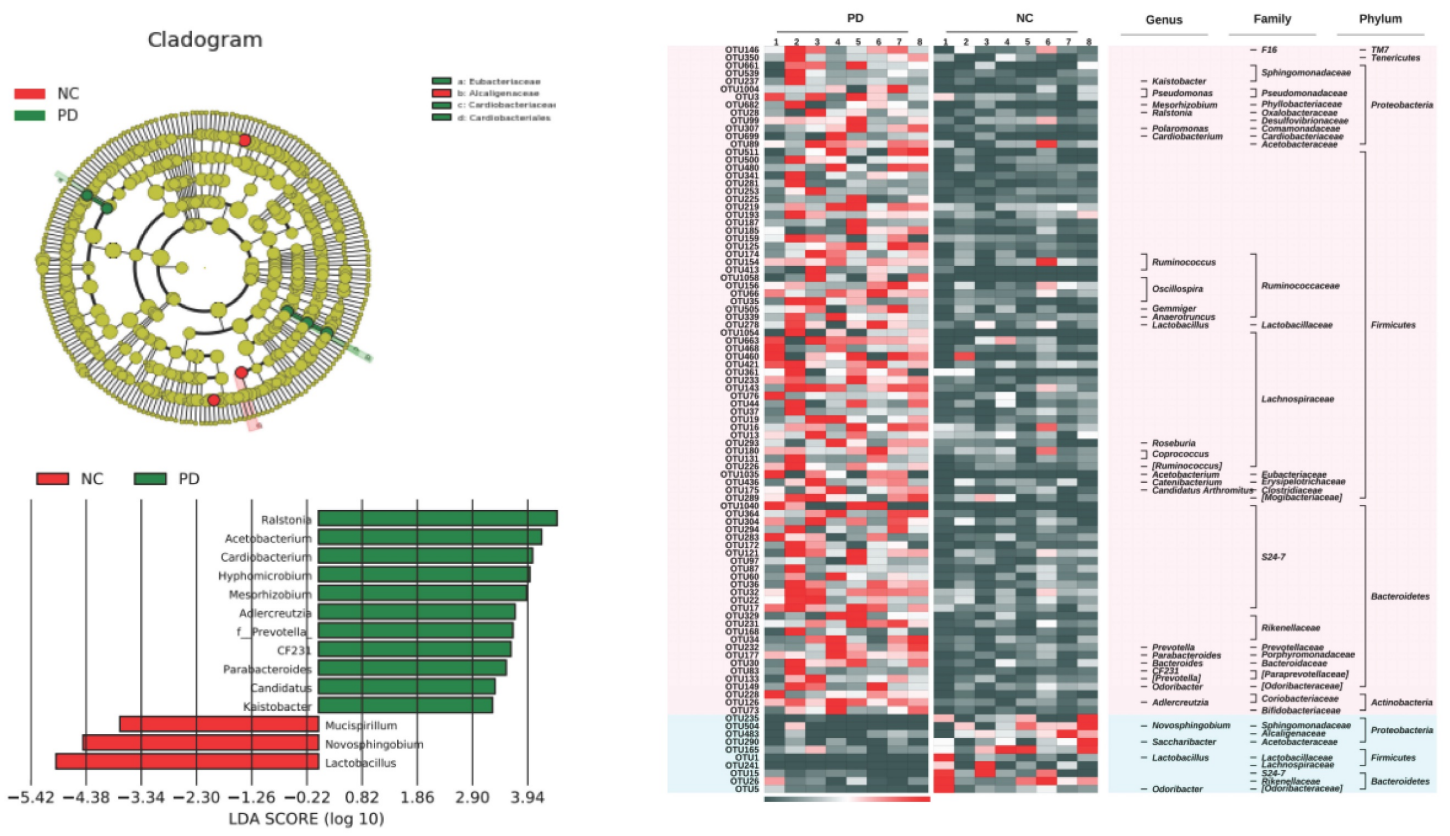
The gut microbe between G1 and G2.

Then, based on LEfSe statistical analysis, differential analysis was conducted on the microbial composition data of the three groups and the results showed that:

Compared with the control group, the TLzmab treatment group showed a decrease in Mucispirium, Novosphingobium, and Lactobacillus, while Ralstonia, Acetobacterium, Cardiobacterium, Hyphomicrobium, Mesorhizobium, Adlercreutzia, Prevotella, CF231, Paraacteroide, Candidatus, and Kaitobacter significantly increased (Figure 5).

**Figure 6:**
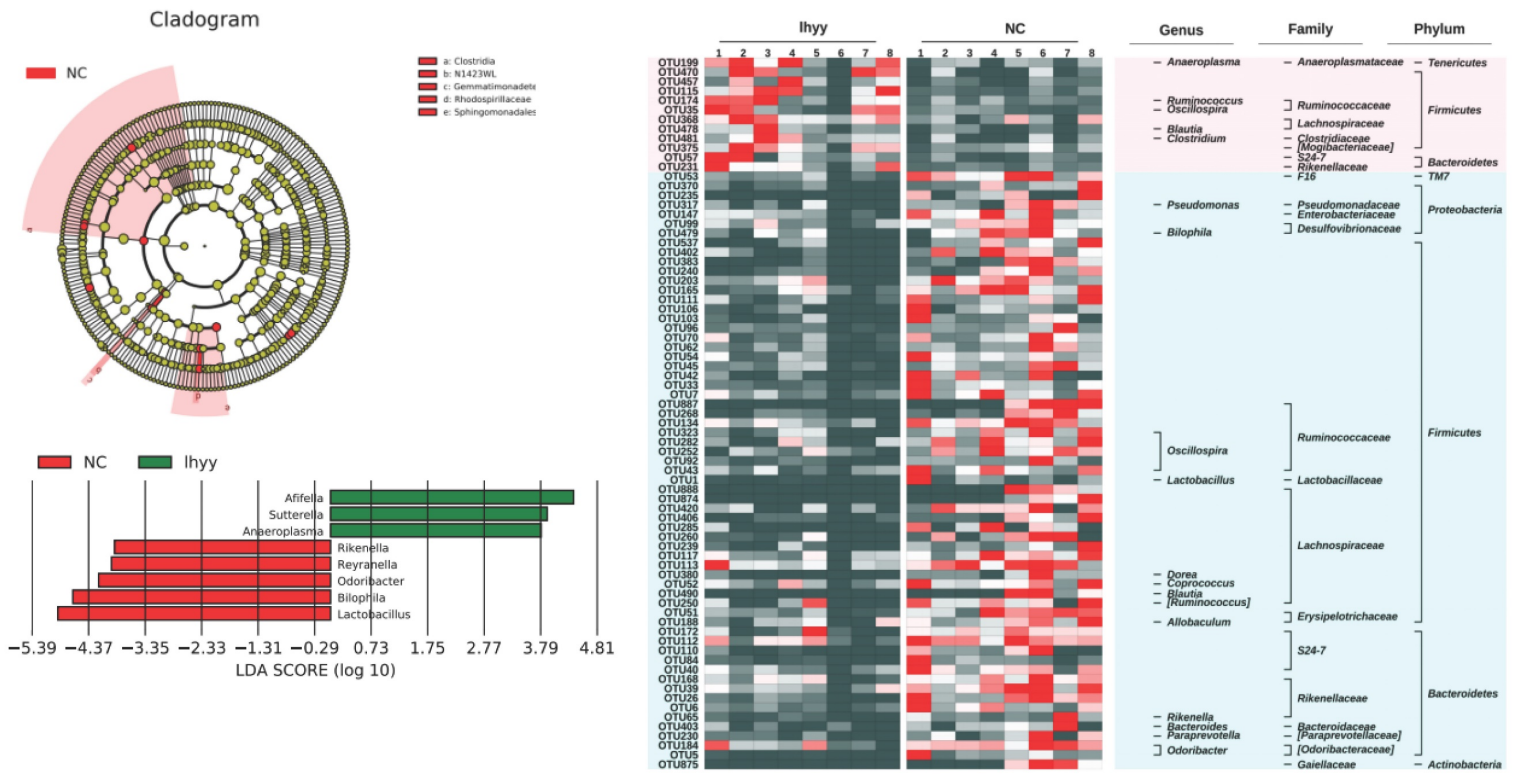
The gut microbe between G1 and G3.

Compared with the control group, the TLzmab+SLBZD group showed a significant decrease in Lactobacillus, Bilaphila, Odoribactor, Reyranella, and Rikenella, while Anaeroplasma, Sutterella, and Afifella increased significantly (Figure 6).

**Figure 7:**
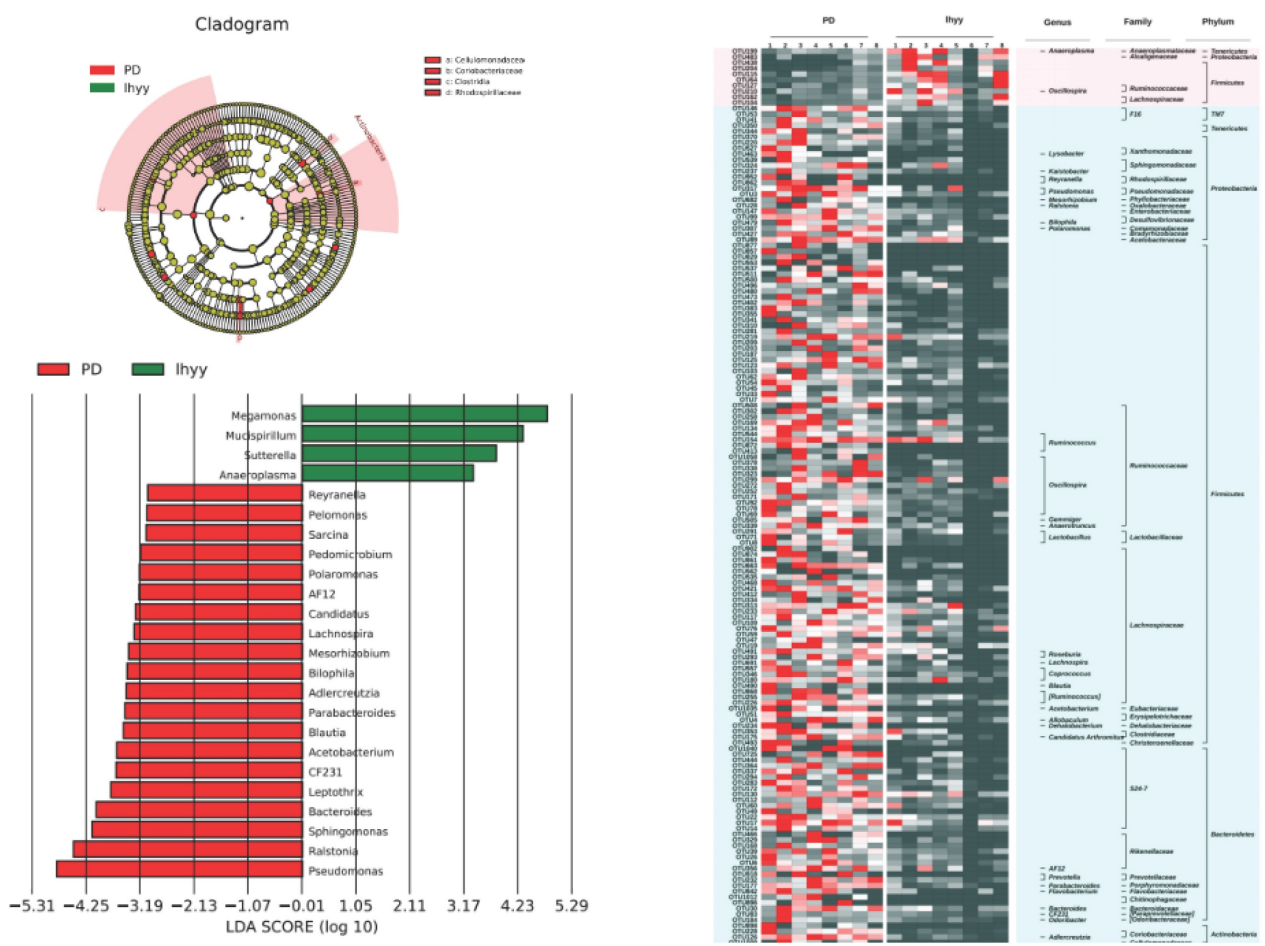
The gut microbe between G2 and G3.

Compared with the TLzmab group, the TLzmab+SLBZD group showed a significant decrease in Pseudomonas, Ralstonia, Sphingomonas, Bacteroides, Leptothrix, CF231, Acetobacterium, Blautia, Paraacteroides, Dercreutzia, Bilaphila, Mesorhizobium, Lachnospira, Candidatus, AF12, Polomonas, Pedomicrobium, Sarcina, Pelomonas, Reyranella, while Anaeroplasma, Sutterella La, ucispirillum, and Megamonas showed a significant increase (Figure 7).

### 3. The effect of SLBZD on the proportion of immune cells in the tumor immune environment

**Figure 8:**
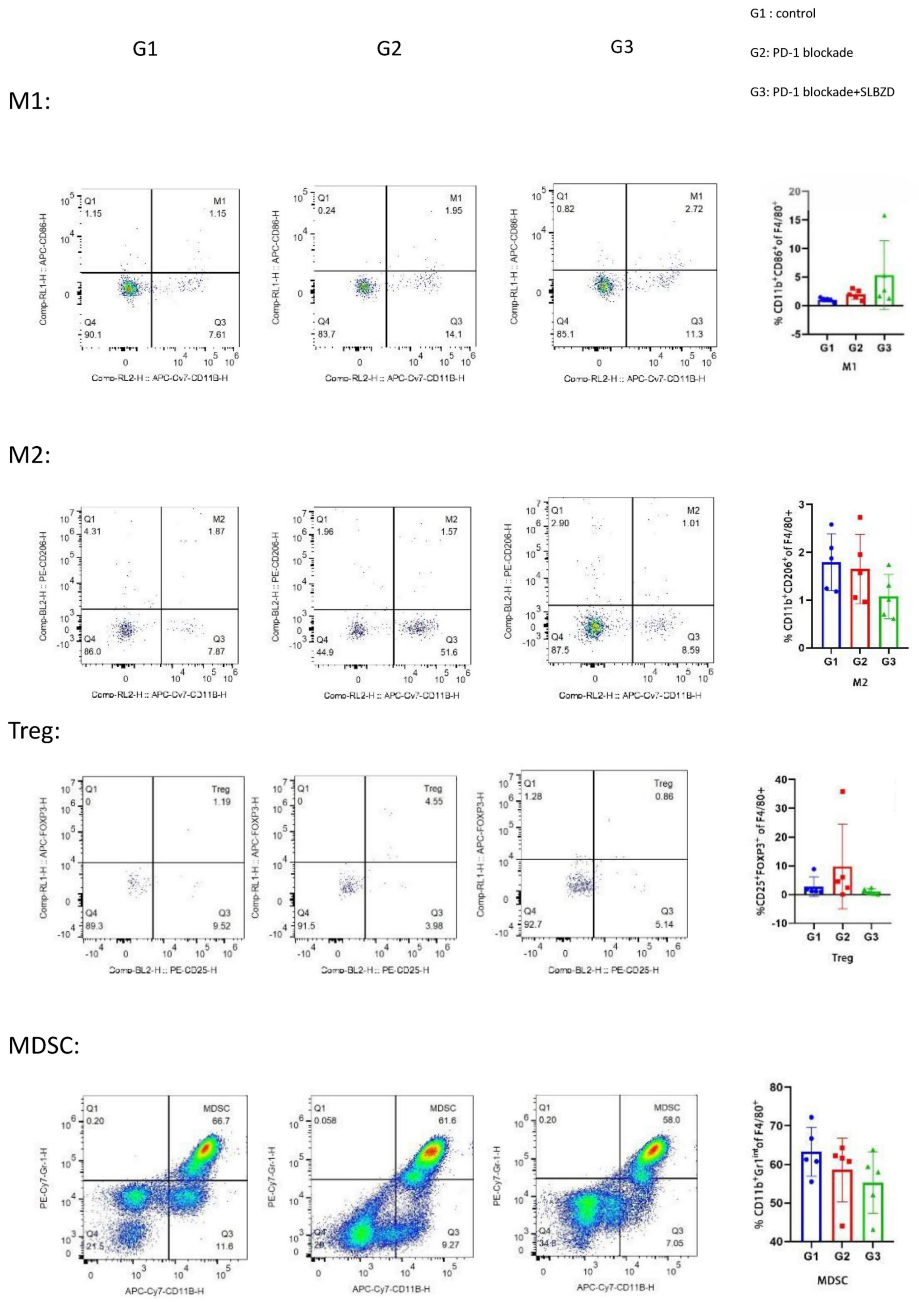
The TME in 3 groups.

The lymphocytes in the peripheral blood of the three groups were stained, and the proportion of lymphocytes in the blood was analyzed by flow cytometry. Compared with the control group, there was no significant difference between the M1 and M2 macrophages in the TLzmab group (p=0.102), while Myeloid-derived suppressor cells(MDSCs) decreased and T regulatory cells (Treg) increased significantly (p<0.001), with a statistically significant difference; Compared with the control group and TLzmab group, the TLzmab+SLBZD group showed a significant increase in M1 type macrophages, while the M2 type macrophages, MDSCs, and Treg showed a significant decrease (p<0.001) (Figure 8).

## Discussion

Although immune checkpoint inhibitors (ICIs) have played an important role in the treatment of colorectal cancer. However, drug resistance and low sensitivity limit their application in the treatment of colorectal cancer. Finding ways that can improve the treatment effect of ICIs and prevent their drug resistance has important clinical value 3-5.

This study investigated whether the traditional Chinese medicine Shenling Baizhu decoction (SLBZD) has the effect of promoting the treatment of immune checkpoint inhibitors. We used the PD-1 inhibitor Letilizumab (TLzmab) to treat a colorectal mouse model and found that TLzmab can inhibit the growth of colorectal cancer tumors. Compared with the control group, the tumor can be significantly inhibited. Encouragingly, after administration of SLBZD, the tumor inhibition rate of TLzmab significantly increased. On the seventh day after the start of the experiment, there was no difference between the TLzmab group and the control group. However, after the combination of SLBZD, the tumor volume was significantly inhibited compared to the control group and TLzmab group, indicating that SLBZD can accelerate the onset time of TLzmab. Moreover, in the observation of 14 and 18 days, the efficacy of the combined SLBZD group was significantly higher than that of the TLzmab alone, with a tumor TGI of more than twice (92.09% vs 39.36%), It is suggested that SLBZD can promote the onset time and tumor inhibition effect of TLzmab monoclonal antibody. Huang *et al.* 16 found that the use of ginseng polysaccharide can also increase the anti-tumor effect of PD-1 inhibitors by improving Gut microbiota. This study found that SLBZD can promote the inhibitory effect of TLzmab on colorectal cancer. There have been no similar reports so far, providing ideas for improving the treatment of tumors with ICIs and a basis for whether similar traditional Chinese medicine can be used to promote ICIs research.

### Why can SLBZD improve the efficacy of TLzmab?

Tian Tingting and others found that the therapeutic effect of SLBZD on CRC may be related to specific biological processes and related pathways to regulate inflammatory response and optimize the structure of Gut microbiota to play a therapeutic role through network pharmacology research 17. In the previous study of this group, it was found that SLBZD and other similar Chinese medicines can improve the imbalance of Gut microbiota and promote the abundance of beneficial intestinal flora 15.

This study found that the use of TLzmab can reduce the diversity of Gut microbiota in experimental mice, while the use of SLBZD can increase the diversity of Gut microbiota, suggesting that SLBZD can play a role in promoting the efficacy of TLzmab in the treatment of colorectal cancer by repairing the diversity of Gut microbiota in TLzmab treatment. Previous studies have also shown that the use of gut microbiota transplantation can improve the efficacy of PD-1 inhibitors 12-14.

From the species level, after TLzmab treatment, the genera of Mucipirillum, Novosphingobium and Lactobacillus in the intestinal tract decreased, while those of Ralstonia, Acetobacterium, Cardiobacterium, Hyphomicrobium, Mesorhizobium, Adlercreutzia, Prevotella, CF231, Parabacteroid, Candidatus and Kaistobacter significantly increased. These gut microbiota play an important role in maintaining Gut microbiota and maintaining mucosal barrier. Muribaculum, also known as Bacteroidales S24-7, has been reported to be related to the efficacy of tumor immunotherapy. Therefore, TLzumab treatment affects the Muribaculum and thus decreases its efficacy 18. Si *et al.* 19 reported dietary lactobacillus-derived exopolyyaccharide enhances immune checkpoint blockade therapy.

Compared with the TLzmab group, the TLzmab+SLBZD group showed a significant decrease in Pseudomonas, Raltonia, Sphingomonas, Bacteroides, Leptothrix, CF231, Acetobacterium, Blautia, Paraacteroides, Dercreutzia, Bilaphila, Mesorhobium, Lachnospira, Candidatus, AF12, Polaromonas, Pedomicrobium, Sarcina, Pelomonas, Reyranella, while An Aeroplasma, Sutterella, ucispirillum Megamonas significantly increased. Anaeroplasma and Sutterella are important symbiotic bacteria in the intestine, which are crucial for maintaining intestinal homeostasis, reducing inflammatory responses, and maintaining intestinal epithelial secretion 20,21. It seems that Aeroplasma, Sutterella, ucispirillum Megamonas can improve the efficacy of immunotherapy, while Adlercreutzia, Prevotella, CF231, *et al.* can reduce the efficacy of immunotherapy.

After SLBZD was used, the abundance and structure of Gut microbiota changed, significantly improving the tumor immune microenvironment. After TLzmab treatment, Treg levels in the tumor immune microenvironment significantly increased. Treg cells are an important negative regulatory factor in ICIs treatment and the main factor leading to treatment failure or drug resistance, similar to previous reports 22. After the addition of SLBZD, M1-type macrophages significantly increased, while M2-type macrophages, MDSCs, and Treg cells significantly decreased. M1-type macrophages are important factors promoting ICIs, while M2-type macrophages and Treg cells are factors that inhibit the efficacy of ICIs 23. Therefore, this result suggests that SLBZD exerts a synergistic effect on TLzmab by increasing M1-type macrophages and inhibiting M2-type macrophages, and Treg cells. MDSCs are factors that inhibit ICIs in the tumor microenvironment 22, which have declined in this group of treatments.

In conclusion, an imbalance of Gut microbiota and imbalance of tumor immune microenvironment will occur during TLzmab treatment, which will lead to poor therapeutic effect of TLzmab or drug resistance. SLBZD will increase the abundance of Gut microbiota and improve the structure of Gut microbiota, which will lead to the increase of M1 macrophages in the tumor immune microenvironment and the decrease of M2 macrophages and Treg cells, thus exerting the synergistic effect of TLzmab. This study provides a new way to explore the improvement of ICIs through traditional Chinese medicine and is worthy of further clinical study.

## Figures and Tables

**Table 1:** The antibodies of immune cells for flow cytometer detection

M1	KIRAVIA Blue 520™ anti-mouse F4/80 Antibody
	APC/Fire™ 750 anti-mouse/human CD11b Antibody
	APC anti-mouse CD86 Antibody
M2	KIRAVIA Blue 520™ anti-mouse F4/80 Antibody
	APC/Fire™ 750 anti-mouse/human CD11b Antibody
	PE anti-mouse CD206 (MMR) Antibody
Treg	PE anti-mouse CD25 Antibody
	Alexa Fluor® 647 anti-mouse FOXP3 Antibody
	FITC anti-mouse CD4 Antibody
MDSC	PE/Cyanine7 anti-mouse Ly-6G/Ly-6C (Gr-1) Antibody
	APC/Fire™ 750 anti-mouse/human CD11b Antibody

**Table 2:** The tumor volume in 3 group (mm^3^)

		Control	TLzmab	SLBZD+TLzmab
0d	M±SD	110.71±9.89	110.53±7.75	114.06±14
7d	M±SD	374.21±40.82	345.49±34.11	197.28±51.97
14d	M±SD	1567.06±333.22	733.96±137.64	206.79±55.7
18d	M±SD	2747.05±589	1665.76±432.39	217.36±61.04
